# The pulsatile component of left atrial pressure has little effect on pulmonary artery impedance estimation in normal rats

**DOI:** 10.14814/phy2.13946

**Published:** 2018-12-16

**Authors:** Masafumi Fukumitsu, Toru Kawada, Shuji Shimizu, Masaru Sugimachi

**Affiliations:** ^1^ Department of Cardiovascular Dynamics National Cerebral and Cardiovascular Center Osaka Japan; ^2^ William Harvey Research Institute Barts and The London School of Medicine Queen Mary University of London London United Kingdom

**Keywords:** Left atrial pressure, One‐input, one‐output system, Pulmonary artery impedance, Two‐input, one‐output system

## Abstract

Pulmonary artery impedance (PAZ) that measures the pulsatile properties of the vasculature provides diagnostic and prognostic information in patients with pulmonary vascular diseases. While downstream pressure [i.e., left atrial (LA) pressure] should be considered when calculating static properties of pulmonary vasculature, PAZ is commonly estimated without taking into account the pulsatile component of LA pressure. We examined whether PAZ can be estimated with reasonable accuracy without using LA pressure. Pulmonary artery (PA) flow, PA pressure, and LA pressure were measured under irregular pacing in eight normal Sprague‐Dawley rats. PAZ was estimated by analyzing a one‐input, one‐output system (I1O1 analysis) that does not include LA pressure, and a two‐input, one‐output system (I2O1 analysis) that includes LA pressure. Using a tube and 3‐element Windkessel model, PAZ was parameterized as peripheral resistance (*R*_P_), arterial compliance (*C*_P_), characteristic impedance (*Z*_C_), and transmission time to the reflection site (*T*_*D*_). These parameters were not significantly different between the I1O1 and I2O1 analyses (*R*_*P*_: 0.286 ± 0.040 vs. 0.274 ± 0.038 mmHg·min/mL, *C*_*P*_: 0.352 ± 0.049 vs. 0.343 ± 0.041 mL/mmHg, *Z*_*C*_: 0.115 ± 0.005 vs. 0.117 ± 0.005 mmHg·min/mL, *T*_*D*_: 13.2 ± 1.8 vs. 12.9 ± 1.7 msec). In conclusion, the I1O1 analysis that does not use LA pressure estimates PAZ with reasonable accuracy compared with the I2O1 analysis that uses LA pressure in normal rats. Our finding that the pulsatile component of LA pressure contributes little to PAZ estimation may justify the clinical use of the I1O1 analysis.

## Introduction

The pressure gradient between upstream and downstream pressures divided by blood flow gives the vasculature resistance (Nichols et al. 2011). In systemic circulation, the downstream pressure (the central venous pressure) is often disregarded when calculating systemic vascular resistance, because the central venous pressure is very low compared with the upstream pressure (the aortic pressure) in normal physiological circumstances. By contrast, in pulmonary circulation, the ratio of mean downstream pressure (left atrial [LA] pressure) to mean upstream pressure (pulmonary artery [PA] pressure) is unignorably high (Bonow et al. 2011; Nichols et al. 2011). In healthy subjects, mean PA and LA pressures are approximately 15 and 8 mmHg, respectively; and thus, mean LA pressure is more than one‐half of mean PA pressure even in normal physiological condition (Bonow et al. 2011). Hence, mean LA pressure can significantly affect vascular resistance calculation. Accordingly, there are two definitions of vascular resistance in pulmonary circulation. One is pulmonary vascular resistance (PVR), which is calculated from the difference between mean PA pressure and mean PA wedge pressure (or LA pressure) divided by mean PA flow. The other is total pulmonary resistance (TPR), which is calculated from mean PA pressure divided by mean PA flow without using LA pressure. TPR is greater than PVR by a value of mean LA pressure divided by mean PA flow. In clinical perspective, mean PA wedge pressure (or LA pressure) should be taken into account when interpreting vascular resistance of the pulmonary circulation (Galiè et al. 2015).

**Figure 1:**
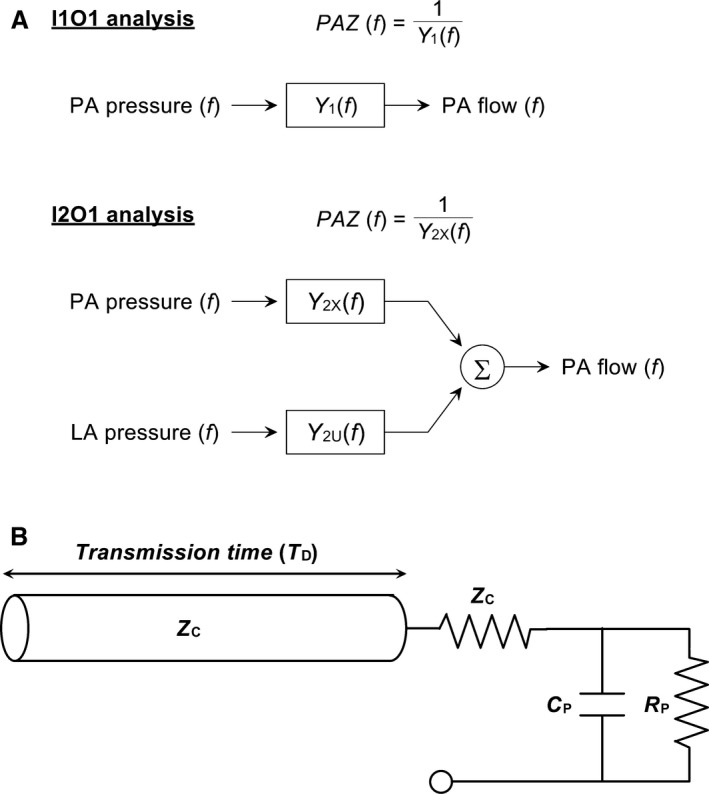
(A) Schemes for one‐input, one‐output (I1O1) analysis and two‐input, one‐output (I2O1) analysis. In the I1O1 analysis, pulmonary artery impedance (PAZ) is calculated as the reciprocal of hydraulic admittance from pulmonary artery (PA) pressure to PA flow [*Y*_1_(*f*)]. In the I2O1 analysis, hydraulic admittance from PA pressure to PA flow [*Y*_2X_(*f*)] and that from left atrial (LA) pressure to PA flow [*Y*_2U_(*f*)] are calculated, and PAZ is determined by the reciprocal of *Y*_2X_(*f*). (B) A schema for an elastic lossless tube with three‐element Windkessel model. The model is characterized by peripheral resistance (*R*_P_), pulmonary arterial compliance (*C*_P_), characteristic impedance (*Z*_C_), and transmission time from an inlet of PA to a reflection site in pulmonary vascular bed (*T*_*D*_).

Both PVR and TPR represent static properties of the pulmonary vasculature in steady‐state flow. On the other hands, dynamic properties of the vasculature in pulsatile flow are described by pulmonary artery impedance (PAZ) (Westerhof et al. 1971). Using a mathematical model, measured PAZ can be parameterized as peripheral resistance, arterial compliance, characteristic impedance, and wave reflection in the pulmonary vascular bed (Lankhaar et al. 2006; Kind et al. 2011; Fukumitsu et al. 2017). Several clinical studies have reported the significance of PAZ parameters in differentiating the etiologies (Lankhaar et al. 2006) and prognosis in patients with pulmonary vascular disease (Mahapatra et al. 2006; Hunter et al. 2008). In clinical studies, PAZ is commonly calculated as a transfer function from PA flow to PA pressure without using LA pressure (Murgo and Westerhof 1984; Huez et al. 2004; Lankhaar et al. 2006; Hunter et al. 2008). PA admittance, which is the reciprocal of PAZ, can be calculated as a transfer function from PA pressure to PA flow (Fig. 1A). Hereinafter, we refer to this analysis as an analysis of one‐input, one‐output system (I1O1 analysis). Alternatively, PA admittance can be calculated by including LA pressure as additional information, in the form of an analysis of two‐input, one‐output system (I2O1 analysis) (Fig. 1A). Theoretically, when two inputs (PA and LA pressure waveforms) are mutually independent, LA pressure does not significantly bias the estimation of PA admittance (Bendat and Piersol 2000) even with the I1O1 analysis. However, the LA pressure waveform may correlate with the PA pressure waveform to a variable degree. With the I2O1 analysis, PA admittance can be obtained in the presence of some correlation between PA and LA pressure waveforms. It is conceivable that the I2O1 analysis should provide more accurate estimation of PA admittance than the I1O1 analysis. However, simultaneous recording of PA and LA pressure waveforms is extremely difficult in clinical settings, which limits the use of the I2O1 analysis. Although PAZ is estimated using the I1O1 analysis in clinical practice, to what extent PAZ estimated by the I1O1 analysis differs from PAZ estimated by the I2O1 analysis remains unknown even in normal physiological circumstances. The aim of the present study was to answer the question of whether PAZ calculated as the reciprocal of PA admittance using the I1O1 analysis is reasonably accurate compared with PAZ calculated as the reciprocal of PA admittance using the I2O1 analysis. To achieve this goal, we used an in vivo experimental technique of estimating PAZ over a wide frequency range of physiological interest (0.1–50 Hz) in rats (Fukumitsu et al. 2016a,b).

## Methods

The animal study was conducted in accordance with the National Institutes of Health Guide for the Care and Use of Laboratory Animals. The experimental protocols were reviewed and approved by the Animal Subjects Committee at the National Cerebral and Cardiovascular Center, Japan.

### Surgical preparation

Experimental data were collected from eight male 12 week‐old Sprague‐Dawley rats. Detailed surgical preparation has been described previously (Fukumitsu et al. 2016a,b). Briefly, the rat was anesthetized with an intraperitoneal injection (2 mL/kg) of α‐chloralose (40 mg/mL) and urethane (250 mg/mL). After tracheotomy, mechanical ventilation was conducted at a rate of 80 breaths/min. The partial pressure of oxygen in arterial blood was maintained at more than 200 mmHg by ventilating with oxygen‐enriched room air. An arterial catheter was placed in the right femoral artery to measure systemic arterial pressure using a fluid‐filled pressure sensor (DX‐200, Nihon‐Kohden, Tokyo, Japan). After a left‐sided thoracotomy, an ultrasound transit time flow probe (MA‐2.5PSB, Transonic Systems, Ithaca, NY, USA) was placed around the main PA. A high‐fidelity catheter‐tipped micromanometer (SPR‐320, Millar Instruments, Houston, TX, USA) was introduced into the main PA through the right ventricular free wall to measure PA pressure, and another micromanometer into the left atrium through the left ventricle (LV) to measure LA pressure. A pair of polyurethane‐coated stainless‐steel wires of 0.08 mm in diameter (Unique Medical, Osaka, Japan) was attached to the LV for electrical pacing.

### Calculation of pulmonary artery impedance

PA pressure, LA pressure, and PA flow were measured simultaneously under sinus rhythm and during irregular pacing. For stable estimation of PAZ, irregular pacing was conducted by changing the RR interval beat by beat according to the following protocol.

(1) $$\text{RR}\;\text{interval}\;\left(\text{msec}\right)=\;50X+100,$$

where *X* is a random number with Gaussian distribution between 0.00 and 0.99 (mean value is 0.49 and standard deviation is 0.28). Data were digitized at 1000 Hz using a 16‐bit analog‐to‐digital converter. The segment for a frequency analysis contained 8192 points, which provided a frequency resolution of 0.122 Hz.

When the I1O1 analysis was used, PAZ [*Z*_*1*_(*f*)] was calculated as the reciprocal of hydraulic admittance from PA pressure to PA flow [*Y*_*1*_(*f*)] [i.e., *Z*_*1*_(*f*) = 1/*Y*_*1*_(*f*), Fig. 1A], and *Y*_*1*_(*f*) was calculated using the following equation.

(2) $$\text{PAF}\left(f\right)=Y_{1}\left(f\right)\cdot \text{PAP}\left(f\right)+N_{\mathrm{PAF}1}\left(f\right)$$

where PAF(*f*) and PAP(*f*) are the Fourier transforms of PA flow and PA pressure, and *N*_PAF1_(*f*) is a noise term in the linear transfer function analysis, respectively. The concordance of PAZ obtained by calculating the reciprocal of *Y*_*1*_(*f*) and PAZ estimated directly as a transfer function from PA flow to PA pressure is affected by the measurement noise (see Appendix 1 for details). However, to focus on the contribution of LA pressure to PAZ estimation by comparing the I1O1 and I2O1 analyses, we adopted the method of calculating the reciprocal of *Y*_*1*_(*f*) in the present study.

When the I2O1 analysis was used, PAZ [*Z*_*2*_(*f*)] was also calculated as the reciprocal of hydraulic admittance from PA pressure to PA flow [*Y*_*2X*_(*f*)] (Fig. 1; see Appendix 2 for details), but *Y*_*2X*_(*f*) was calculated using an equation that includes the effect of LA pressure on PA flow, as follows.

(3) $$\begin{array}{cc} \text{PAF}\left(f\right)= & \;Y_{2X}\left(f\right)\cdot \text{PAP}\left(f\right)+Y_{2U}\left(f\right)\cdot \text{LAP}\left(f\right)\\ & +N_{\mathrm{PAF}2}\left(f\right) \end{array}$$

where LAP(*f*) and *Y*_*2U*_(*f*) are the Fourier transforms of LA pressure and hydraulic admittance from LA pressure to PA flow, and *N*_PAF2_(*f*) is a noise term in the linear transfer function analysis, respectively.

Using the transfer function from PA pressure to LA pressure [*H*_*3*_(*f*)], the relation among *Y*_*1*_(*f*), *Y*_*2X*_(*f*), and *Y*_*2U*_(*f*) can be expressed as follows (see Appendix 3 for details).

(4) $$Y_{1}\left(f\right)=Y_{2X}\left(f\right)+Y_{2U}\left(f\right)\cdot H_{3}\left(f\right)$$

After estimating PAZ by both I1O1 and I2O1 analyses, each PAZ was parameterized using a model consisting of a tube and the 3‐element Windkessel model (tube‐3WK model) as shown in Figure 1B. The parameters of the model are peripheral resistance (*R*_P_), pulmonary arterial compliance (*C*_P_), characteristic impedance (*Z*_C_), and transmission time from the proximal artery to the reflection site (*T*_*D*_) in the pulmonary vascular bed (Sugimachi et al. 1997; Fukumitsu et al. 2017). These parameters were determined by fitting a tube‐3WK model to the measured data using an iterative nonlinear least‐square method (Nelder and Mead 1965) for the frequency range between 0.122 and 48.8 Hz (400 points) to minimize the following error function.

(5) $$\begin{array}{cc} \text{error}= & \sum_{k=1}^{400}\frac{{\left|\mathrm{lo}g_{10}\left[Z_{\mathrm{est}}\left(f\right)\right]-\mathrm{lo}g_{10}\left[Z_{\mathrm{model}}\left(f\right)\right]\right|}^{2}}{k},\;\;\\ & f=f_{0}\times k, \end{array}$$

where *Z*_est_(*f*) and *Z*_model_(*f*) are the estimated PAZ and model PAZ, respectively; *k* is the frequency index; and *f*_0_ is the fundamental frequency (0.122 Hz).

Baseline hemodynamic parameters including heart rate, systemic arterial pressure, PA and LA pressures, and PA flow were averaged over 1 min under normal sinus rhythm without pacing.

### Quantification of difference between I1O1 and I2O1 analyses

The differences between *Z*_*1*_(*f*) and *Z*_*2*_(*f*) at each frequency were evaluated by calculating the modulus difference expressed in common logarithm [*R*_modulus_(*f*)] and the phase difference [D_phase_(*f*)] as follows.

(6) $$R_{\mathrm{modulus}}\left(f\right)=\log_{10}\frac{\left|Z_{1}\left(f\right)\right|}{\left|Z_{2}\left(f\right)\right|}$$

(7) $$D_{\mathrm{phase}}\left(f\right)=\tan^{-1}\left[\frac{Z_{1}\left(f\right)}{Z_{2}\left(f\right)}\right]$$

### Statistical analysis

All data are expressed as mean ± standard error. Parameters of PAZs estimated by the I1O1 and I2O1 analyses were compared using paired t‐test or Wilcoxon signed rank test, following Kolmogorov–Smirnov test. Correlation of the modulus (expressed in common logarithm) of *Z*_*1*_(*f*) versus modulus of *Z*_*2*_(*f*) for the frequency range of 0.122–48.8 Hz was examined by Pearson correlation test. Correlation of the phase of *Z*_*1*_(*f*) versus phase of *Z*_*2*_(*f*) was likewise analyzed. A total of 3200 points (400 points per rat) were used for the correlation analysis. *P* values less than 0.05 were considered statistically significant.

## Results

**Figure 2:**
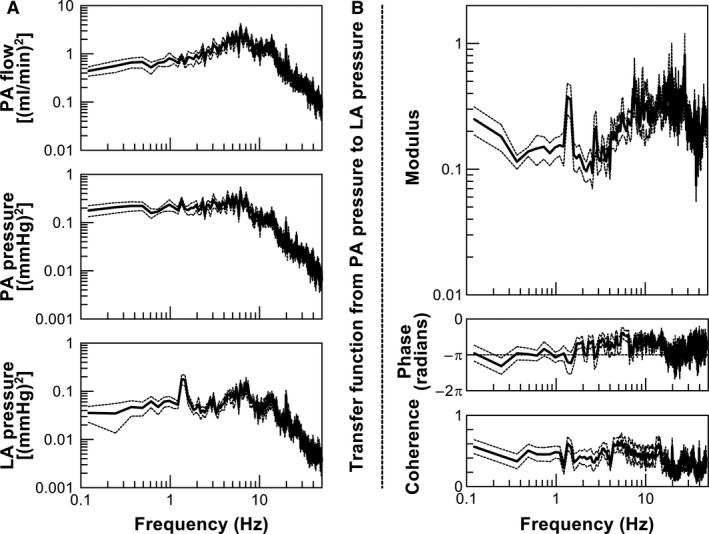
(A) Averaged power spectra of pulmonary artery (PA) flow, PA pressure, left atrial (LA) pressure. (B) Averaged modulus and phase of a transfer function from PA pressure to LA pressure, and coherence function. Solid lines denote averaged data, and dotted lines denote mean ± standard error.

Body weight and hemodynamic parameters under normal sinus rhythm are shown in Table 1. The power spectra of PA flow, PA pressure, and LA pressure during irregular pacing are shown in Figure 2A. The ratio of square root of power spectrum of LA pressure to that of PA pressure in the frequency range of 0.1–1 Hz was significantly lower than the ratio of mean LA pressure to PA pressure (Table 2).

**Table 1:** Body weight and hemodynamic parameters of rats under normal sinus rhythm

	Normal (*n* = 8)
Body weight, g	422 ± 10
HR, beats/min	388 ± 14
Mean SAP, mmHg	77.9 ± 5.9
Mean PA flow, mL/min	26.9 ± 3.8
Mean PA pressure, mmHg	14.2 ± 0.6
Pulsatility of PA pressure, mmHg	24.3 ± 1.43
Mean LA pressure, mmHg	7.3 ± 0.7
Pulsatility of LA pressure, mmHg	11.5 ± 1.54
Ratio of mean LA pressure to mean PA pressure	0.51 ± 0.38
Ratio of pulsatility of LA pressure to that of PA pressure	0.50 ± 0.09
TPR, mmHg·min/mL	0.60 ± 0.08
PVR, mmHg·min/mL	0.30 ± 0.07

Data are expressed as mean ± standard error. HR, heart rate; SAP, systemic arterial pressure; PA, pulmonary artery; LA, left atrium; Pulsatility of pressure was determined by the difference between the maximum and the minimum values of pressure. TPR, total pulmonary resistance calculated by PA pressure/PA flow, PVR, pulmonary vascular resistance calculated by (PA pressure ‐ LA pressure)/PA flow.

**Table 2:** Pulmonary artery and left atrial pressure power spectra under irregular pacing

	Normal (*n* = 8)
PA pressure power spectrum, (mmHg)^2^	
0.1–1 Hz	0.20 ± 0.03
1–10 Hz	0.22 ± 0.02
10–50 Hz	0.04 ± 0.002
LA pressure power spectrum, (mmHg)^2^	
0.1–1 Hz	0.05 ± 0.01
1–10 Hz	0.07 ± 0.01
10–50 Hz	0.02 ± 0.003
Ratio of square root of power spectrum of LA pressure to that of PA pressure	
0.1–1 Hz	0.39 ± 0.03^a^
1–10 Hz	0.48 ± 0.04
10–50 Hz	0.63 ± 0.04

Data are expressed as mean ± standard error.

PA, pulmonary artery; LA, left atrium.

a *P* < 0.05 versus the ratio of mean LA pressure to mean PA pressure by Friedman test followed by Wilcoxon signed rank test with Bonferroni post hoc correction.

Figure 2B shows the modulus and phase of *H*_*3*_(*f*), and the corresponding coherence function. The modulus of *H*_*3*_(*f*) varies from 0.1 to 0.4, with peaks at the ventilation frequency (approximately 1.3 Hz) and its harmonics in the frequency range below approximately 7 Hz. Above 7 Hz, the modulus of *H*_*3*_(*f*) increased to approximately 0.6 with fluctuation. The phase was around –*π* in the frequency range below approximately 2 Hz, and increased toward zero but remained negative in the frequency range between approximately 2 and 50 Hz. The coherence function between PA and LA pressures showed relatively constant (approximately 0.3–0.6) up to 15 Hz, and it was less than 0.3–0.4 with fluctuation above 15 Hz.

**Figure 3:**
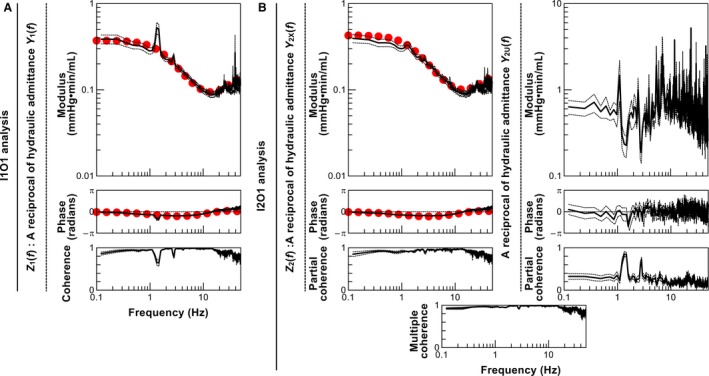
(A) Averaged modulus and phase of pulmonary artery impedance (PAZ) estimated by one‐input, one‐output (I1O1) analysis [Z_*1*_(*f*)], and coherence function. (B) Averaged modulus and phase of PAZ estimated by two‐input, one‐output (I2O1) analysis [Z_*2*_(*f*)], and partial coherence function (left panels). Averaged modulus and phase of the reciprocal of hydraulic admittance from LA pressure to PA flow [*Y*_*2U*_(*f*)], and partial coherence function (right panels). Multiple coherence function between the combination of two inputs (PA and LA pressure) and one output (PA flow) is demonstrated in the bottom panel. Solid lines denote averaged data, and dotted lines denote mean ± standard error. Red dotted lines denote a tube and three‐element Windkessel model fitted to averaged modulus and phase of PAZ estimated by I1O1 or I2O2 analyses.

Figure 3A and B summarize the moduli and phases of *Z*_*1*_(*f*) (Fig. 3A) and *Z*_*2*_(*f*) (left panels in Fig. 3B) and the corresponding coherence functions. The modulus showed the same trend in both *Z*_*1*_(*f*) and *Z*_*2*_(*f*); showing relatively constant values (0.3–0.4 mmHg·min/mL) up to a frequency of around 1 Hz, decreasing thereafter until 10 Hz, and again becoming relatively constant (0.08–1.0 mmHg·min/mL) above 10 Hz. However, *Z*_*1*_(*f*), but not *Z*_*2*_(*f*), showed a high peak at the ventilation frequency (approximately 1.3 Hz). In both *Z*_*1*_(*f*) and *Z*_*2*_(*f*), the phase was close to zero radians at the lowest frequency, and was slightly delayed in the frequency range of 0.4–10 Hz, followed by a slight advance beyond 20 Hz. An irregular phase change was noted in *Z*_*1*_(*f*) at the ventilation frequency. While the partial coherence function associated with *Z*_*2*_(*f*) remained close to unity up to 30 Hz, the coherence function associated with *Z*_*1*_(*f*) dropped to approximately 0.6 at the ventilation frequency and again to 0.8 at its second harmonic. Both *Z*_*1*_(*f*) and *Z*_*2*_(*f*) were parameterized using a tube‐3WK model. As shown in Table 3, R_*P*_, *C*_*P*_, *Z*_*C*_, and *T*_*D*_ were not significantly different between the I1O1 and I2O1 analyses.

**Table 3:** Parameters of pulmonary artery impedance (PAZ) calculated from an analysis of a one‐input, one‐output system (I1O1 analysis) and an analysis of a two‐input, one‐output system (I2O1 analysis)

	I1O1 analysis	I2O1 analysis	*P* value
*R*_*P*_, mmHg·min/mL	0.286 ± 0.040	0.274 ± 0.038	0.476
*C*_*P*_, mL/mmHg	0.352 ± 0.049	0.343 ± 0.041	0.582
*Z*_*C*_, mmHg·min/mL	0.115 ± 0.005	0.117 ± 0.005	0.103
*T*_*D*_ *,* msec	13.2 ± 1.8	12.9 ± 1.7	0.359

Data are expressed as mean ± standard error. *R*_P_, peripheral resistance, *C*_P_, pulmonary artery compliance, *Z*_C_, characteristic impedance, *T*_D_, transmission time in the tube and 3‐element Windkessel model fitted to measured pulmonary artery impedance. *P* values were calculated using Wilcoxon signed rank test for paired data.

Results of the reciprocal of hydraulic admittance from LA pressure to PA flow, 1/*Y*_*2U*_(*f*), are depicted in Fig. 3B (right panels). The modulus was approximately 0.6–0.7 mmHg·min/mL up to 1 Hz, and varied considerably at frequencies above 1 Hz. The phase was near zero radians at the lowest frequency, and the variation increased at frequencies above 1 Hz. The partial coherence function was less than 0.4 in the frequency range analyzed, except peaks at the ventilation frequency and its second harmonic.

**Figure 4:**
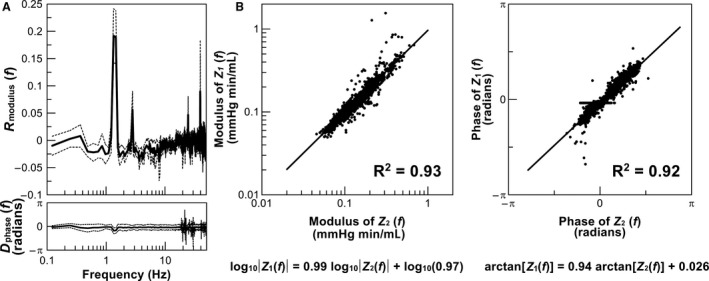
(A) Averaged data showing the difference between pulmonary artery impedance (PAZ) estimated by one‐input, one‐output analysis (I1O1) and PAZ estimated by two‐input, one‐output analysis (I2O1). Difference in modulus was calculated as the difference from the PAZ modulus by I1O1 analysis expressed in the common logarithm to that by I2O1 analysis. Difference in phase is the difference of the PAZ phase by I1O1 analysis from that by I2O1 analysis. Solid lines denote averaged data, and dotted lines denote mean ± standard error. (B) Correlation of modulus (left panel; expressed in common logarithm) and phase (right panel) of PAZ estimated by I1O1 analysis [Z_*1*_(*f*)] with those estimated by I2O1 analysis [Z_*2*_(*f*)]. Coefficients of determination are demonstrated in each panel.

Figure 4A illustrates the differences between *Z*_*1*_(*f*) and *Z*_*2*_(*f*) for the entire frequency range. *R*_modulus_(*f*) was distributed from −0.025 to 0.025 over a wide range of frequency, except peaks at the ventilator frequency of 1.3 Hz and its second harmonic. *D*_phase_(*f*) was near zero radians in the frequency range up to 20 Hz. As shown in Figure 4B, the common logarithm of *Z*_*1*_(*f*) modulus and that of *Z*_*2*_(*f*) modulus demonstrated a linear relationship with a slope of 0.99 (coefficient of determination, R^2^ = 0.93). A high correlation between *Z*_*1*_(*f*) phase and *Z*_*2*_(*f*) phase was also observed (R^2^ = 0.92), with a regression slope of 0.94.

## Discussion

The present study is the first to quantify the difference between PAZs estimated by the I1O1 and I2O1 analyses under irregular pacing in normal rats. Although the I2O1 analysis was more robust than the I1O1 analysis in estimating PAZ at the ventilator frequency and its harmonics, there were no significant differences in fitted parameters between *Z*_*1*_(*f*) and *Z*_*2*_(*f*) (Table 3).

Clinically, the downstream pressure (mean LA pressure or PA wedge pressure) should be taken into consideration in estimating vascular resistance of the pulmonary circulation. However, the I1O1 analysis that does not include LA pressure is commonly used for estimating PAZ. When PA and LA pressures are mutually independent, LA pressure waveform does not affect PAZ estimation. However, the significant correlation between LA and PA pressure could be observed over the wide frequency range (Fig. 2B). As shown in Equation (4), the accuracy of PAZ calculated by the I1O1 analysis depends on *Y*_*2U*_(*f*) and *H*_*3*_(*f*) (the transfer function from PA to LA pressure waveform) (see Appendix 3 for details). Hence, LA pressure can bias the PAZ estimation at frequencies where the modulus of *H*_*3*_(*f*) is large. The I2O1 analysis that takes LA pressure into consideration should be more robust than the I1O1 analysis. As expected, *Z*_*1*_(*f*) (I1O1 analysis), but not *Z*_*2*_(*f*) (I2O1 analysis), showed significant variations at the ventilation frequency and its harmonics (Fig. 3A and B). Such variations of impedance values in *Z*_*1*_(*f*) do not likely reflect true variations of vascular impedance. The moduli at these frequencies were overestimated in *Z*_*1*_(*f*) compared with *Z*_*2*_(*f*) (Fig. 4A). Therefore, the I2O1 analysis might improve PAZ estimation in the frequencies related to ventilation frequency and its harmonics, where the modulus of *H*_*3*_(*f*) is large. Nevertheless, when PAZ was characterized using a tube‐3WK model, the fitted parameters were not significantly different between *Z*_*1*_(f) and *Z*_*2*_(*f*) (Table 3).

In the 3WK model, regardless of the inclusion of a tube component, the modulus asymptotically approaches *Z*_*C*_ + *R*_*P*_ as frequency decreases toward zero. On the other hand, the modulus asymptotically approaches *Z*_*C*_ as frequency increases toward infinity. The modulus in the higher frequency range may show oscillations before reaching *Z*_*C*_ in the tube‐3WK model. As these characteristics are mainly determined from PAZ in the frequency ranges below 1 Hz and above 10 Hz (Fig. 3A), the presence of ventilation‐related variations in *Z*_*1*_(*f*) may not significantly affect the estimates of *Z*_*C*_ and *R*_*P*_. Furthermore, by fitting the model in the frequency range of 0.122–48.8 Hz, the remaining parameters (*C*_*P*_ and *T*_*D*_) can be stably estimated according to the overall shape of the PAZ, without significant influence from ventilation‐related variations in *Z*_*1*_(*f*). Our finding on little effect of LA pressure on PAZ estimation was also observed when a simple 3WK model, one of the most established arterial model, was applied to PAZ parameterization (data not shown). Hence, PAZ may be estimated by the I1O1 analysis with reasonable accuracy compared with the I2O1 analysis.

While PAZ was calculated under irregular pacing in the present study, our finding could be applicable to PAZ calculation under normal sinus rhythm, which is the method commonly used in the clinical setting (Huez et al. 2004; Lankhaar et al. 2006; Hunter et al. 2008). When PAZ was estimated under normal sinus rhythm, there were no significant differences of PAZ modulus and phase around the frequency of heart rate between the I1O1 and I2O1 analyses (modulus: 0.115 ± 0.006 vs. 0.116 ± 0.006 mmHg·min/mL, phase: −0.484 ± 0.059 vs. −0.538 ± 0.071 radians).

### Limitations

First, positive pressure ventilation was used in the present study. Unlike spontaneous respiration, mechanical ventilation with high tidal volume potentially increases PVR and *Z*_*C*_ (Murgo and Westerhof 1984; Cheifetz et al. 1998). In addition, the intrathoracic pressure was equal to atmospheric pressure because of the thoracotomy. These experimental settings may have affected the relationship among alveolar, PA, and LA pressures, which could influence the PAZ estimation (19). Further experiments are required to quantify the effects of ventilatory parameters on PAZ.

Second, irregular electrical pacing from LV was conducted in the present study. LV pacing exerts an electrical dissociation between LA and RV, which might alter the coherence function between PA and LA pressures in the wide range of frequency excepting the LA contraction frequency (corresponding to the native heart rate frequency, i.e., approximately 5–7 Hz) and its harmonics. Although little effect of LA pressure on PAZ estimation was confirmed under normal sinus rhythm, further investigation under irregular pacing from the atrium would be important to examine the difference between the I1O1 and I2O1 analyses under the condition with normal electrical coupling between the atria and ventricles.

Third, the present study was conducted in normal physiological condition. As LA pressure waveform can affect PAZ estimation via *Y*_*2U*_(*f*) and *H*_*3*_(*f*), there is increasing interest on changes of *Y*_*2U*_(*f*) and *H*_*3*_(*f*) in the pathological conditions. For the robust application of the present findings in the clinical settings, further studies will be required to determine whether PAZ estimated by the I1O1 analysis concords with that estimated by the I2O1 analysis in the pathological conditions.

## Conclusion

In contrast to the static, direct current component of LA pressure that discriminates PVR from TPR, the pulsatile alternating current component of LA pressure contributes little to PAZ estimation in normal rats. The I1O1 analysis that does not include the pulsatile component of LA pressure estimates PAZ with reasonable concordance with the I2O1 analysis, provided that PAZ is parameterized with a tube‐3WK model. The present study may provide a rationale for the PAZ estimation using the I1O1 analysis, and will increase the opportunity to measure PAZ in clinical settings.

## Conflict of Interest

none

## Figures and Tables

**Figure A1:**
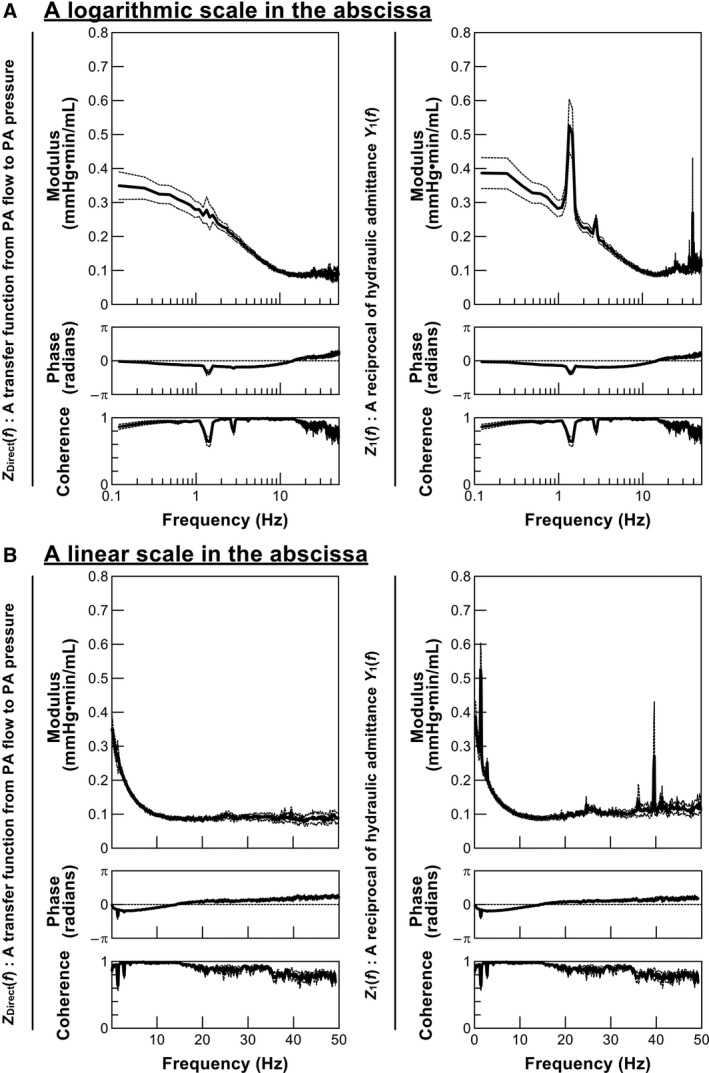
Averaged data of modulus and phase of pulmonary artery impedance estimated directly as a transfer function from PA flow to PA pressure [Z_*Direct*_(*f*)] and the data calculated as the reciprocal of hydraulic admittance from PA pressure to PA flow [Z_*1*_(*f*)], and the corresponding coherence functions. Solid lines denote averaged data, and dotted lines denote mean ± standard error. (A) In the upper panels, data are plotted on a logarithmic scale in the abscissa. (B) In the lower panels, data shown in the upper panels are duplicated on a linear scale in the abscissa to better illustrate the differences in the high frequency range.

## Appendix 1 Comparison between PAZ calculated as a reciprocal of hydraulic admittance and that obtained directly as a transfer function from PA flow to PA pressure

In an ideal linear system without noise, PAZ [Z_*1*_(*f*)] calculated as the reciprocal of hydraulic admittance, that is, the transfer function from PA pressure to PA flow, is equal to PAZ estimated directly as a transfer function from PA flow to PA pressure [Z_Direct_(*f*)]. Given the presence of noise in physiological measurements and system nonlinearity, however, Z_Direct_(*f*) may not be identical to Z_*1*_(*f*). Figure A1 demonstrates the averaged data of modulus and phase of Z_Direct_(*f*) and Z_*1*_(*f*), and the corresponding coherence function in eight normal rats. As shown in Figure A1A, the modulus of Z_Direct_(*f*) is lower than that of Z_*1*_(*f*) in the frequency range of 0.1–1 Hz, at which coherence function decreases slightly to 0.8–0.9. Above 1 Hz, Z_Direct_(*f*) generally agrees with Z_*1*_(*f*) at frequencies up to 15 Hz, at which the coherence function is close to unity, except the peaks observed at ventilatory frequency and its harmonics. However, as demonstrated in Figure A1B, the coherence function tends to decrease at frequencies above 15 Hz, and the modulus of Z_Direct_(*f*) is lower than that of Z_*1*_(*f*). When Z_Direct_(*f*) and Z_*1*_(*f*) are quantified using a tube‐3WK model, *R*_*P*_ and *Z*_*C*_ estimated by Z_Direct_(*f*) are significantly smaller than those estimated by Z_*1*_(*f*) [*R*_*P*_ estimated by Z_Direct_(*f*), 0.252 ± 0.03 mmHg min/ml; *Z*_*C*_ estimated by Z_Direct_(*f*), 0.103 ± 0.01 mmHg min/ml; *P* < 0.05 for both]. In the present study, we estimated PAZ using Z_*1*_(*f*), the reciprocal of hydraulic admittance, by I1O1 analysis to evaluate whether the pulsatile component of LA pressure significantly affects PAZ estimation.

## Appendix 2 PAZ estimations by I1O1 and I2O1 analyses

The signals of PA pressure, PA flow, and LA pressure were segmented into half‐overlapping 12 segments with a length of 8192 points (8.192 s). In each segment, the linear trend was removed and a Hanning window was applied. The input powers of PA pressure [*S*_*XX*_(*f*)] and LA pressure [*S*_*UU*_(*f*)], the output power of PA flow [*S*_*YY*_(*f*)], and the cross‐spectra between combinations of them [*S*_*YX*_(*f*), *S*_*YU*_(*f*), *S*_*XU*_(*f*)] were calculated via the fast Fourier transform and ensemble averaged over the 12 segments.

In a one‐input, one‐output analysis, hydraulic admittance is calculated as a transfer function from PA pressure to PA flow [*Y*_*1*_(*f*)] using the following equation (Bendat and Piersol 2000; Fukumitsu et al. 2016a).

(A1) $$Y_{1}\left(f\right)=\frac{S_{\mathrm{YX}}\left(f\right)}{S_{\mathrm{XX}}\left(f\right)}$$

Pulmonary artery impedance [*Z*_*1*_(*f*)] is then calculated as a reciprocal of *Y*_*1*_(*f*) as follows,

(A2) $$Z_{1}\left(f\right)=\frac{1}{Y_{1}\left(f\right)}$$

The magnitude‐squared coherence function, which is a measure of linear dependence between two signals at each frequency, is calculated as follows.

(A3) $$\text{Coh}\left(f\right)=\frac{{\left|S_{\mathrm{YX}}\left(f\right)\right|}^{2}}{S_{\mathrm{XX}}\left(f\right)S_{\mathrm{YY}}\left(f\right)}$$

A two‐port analysis is used to fully describe the relationships among the upstream pressure and flow and the downstream pressure and flow as follows.

(A4) $$\left[\begin{array}{c} PAF\left(f\right)\\ PVF\left(f\right) \end{array}\right]=\left[\begin{array}{cc} Y_{2X}\left(f\right) & Y_{2U}\left(f\right)\\ Y_{2V}\left(f\right) & Y_{2W}\left(f\right) \end{array}\right]\left[\begin{array}{c} PAP\left(f\right)\\ PVP\left(f\right) \end{array}\right]$$

where *PAF*(*f*), *PAP*(*f*), *PVF*(*f*), *PVP*(*f*) represent the Fourier transforms of PA flow, PA pressure, pulmonary venous flow, and pulmonary venous pressure, respectively; and *Y*_*2X*_(*f*), *Y*_*2U*_(*f*), *Y*_*2V*_(*f*), and *Y*_*2W*_(*f*) are the Fourier transforms of hydraulic admittance between combinations of flow and pressure signals. While pulmonary venous pressure is the same as LA pressure, information on pulmonary venous flow was not obtained in the present study. Hence, we only examined the relationships among *PAF*(*f*), *PAP*(*f*), and *LAP*(*f*) in the form of a two‐input, one‐output analysis, as described in Equation (3) in the main text.

In the two‐input, one‐output analysis, hydraulic admittance is determined by the transfer function from PA pressure to PA flow [*Y*_*2X*_(*f*)], using the following equations (Kawada et al. 1997; Bendat and Piersol 2000).

(A5) $$S_{\mathrm{YXU}}\left(f\right)=S_{\mathrm{YX}}\left(f\right)-\frac{S_{\mathrm{YU}}\left(f\right)}{S_{\mathrm{UU}}\left(f\right)}\cdot S_{\mathrm{UX}}\left(f\right)$$

(A6) $$S_{\mathrm{XXU}}\left(f\right)=S_{\mathrm{XX}}\left(f\right)-\frac{S_{\mathrm{XU}}\left(f\right)}{S_{\mathrm{UU}}\left(f\right)}\cdot S_{\mathrm{UX}}\left(f\right)$$

(A7) $$Y_{2X}\left(f\right)=\frac{S_{\mathrm{YXU}}\left(f\right)}{S_{\mathrm{XXU}}\left(f\right)},$$

where *S*_*UX*_(*f*) is the complex conjugate of *S*_*XU*_(*f*). *Z*_*2*_(*f*) is a reciprocal of *Y*_*2X*_(*f*) as follows.

(A8) $$Z_{2}\left(f\right)=\frac{1}{Y_{2X}\left(f\right)}$$

The hydraulic admittance from LA pressure to PA flow [*Y*_*2U*_(*f*)] can be calculated by exchanging *X* and *U* in the above equation. The magnitude‐squared partial coherence function from PA pressure to PA flow [*Coh*_*X*_(*f*)] is estimated using the following equations.

(A9) $$S_{\mathrm{YYU}}\left(f\right)=S_{\mathrm{YY}}\left(f\right)-\frac{S_{\mathrm{YU}}\left(f\right)}{S_{\mathrm{UU}}\left(f\right)}\cdot S_{\mathrm{UY}}\left(f\right)$$

(A10) $${\text{Coh}}_{X}\left(f\right)=\frac{{\left|S_{\mathrm{YXU}}\left(f\right)\right|}^{2}}{S_{\mathrm{XXU}}\left(f\right)S_{\mathrm{YYU}}\left(f\right)},$$

where *S*_*UY*_(*f*) is the complex conjugate of *S*_*YU*_(*f*). The magnitude‐squared multiple coherence between the combination of two inputs (PA pressure and LA pressure) and one output (PA flow) is calculated using the following equation.

(A11) $${\text{Coh}}_{\mathrm{mul}}\left(f\right)=\frac{Y_{2X}\left(f\right)S_{\mathrm{XY}}\left(f\right)+Y_{2U}\left(f\right)S_{\mathrm{UY}}\left(f\right)}{S_{\mathrm{YY}}\left(f\right)},$$

where *S*_*XY*_(*f*) is the complex conjugate of *S*_*YX*_(*f*).

## Appendix 3 Effect of a transfer function from PA to LA pressure [*H*_*3*_(*f*)] on PA admittance estimation by I1O1 analysis

With the I2O1 analysis, PA flow can be determined by

(3) $$\text{PAF}\left(f\right)=Y_{2X}\left(f\right)\cdot \text{PAP}\left(f\right)+Y_{2U}\left(f\right)\cdot \text{LAP}\left(f\right)+N_{\mathrm{PAF}2}\left(f\right)$$

When LA pressure correlates with PA pressure, *LAP*(*f*) is given as follows.

(A12) $$\text{LAP}\left(f\right)=H_{3}\left(f\right)\cdot \text{PAP}\left(f\right)+{\text{LAP}}_{0}\left(f\right)+N_{\mathrm{LAP}}\left(f\right),$$

where LAP_0_(*f*) is a component of LA pressure waveform independent of PAP(*f*), and *N*_LAP_(*f*) is a noise term. Using Equation A12, Equation (3) states as follows.

(A13) $$\text{PAF}\left(f\right)=Y_{2X}\left(f\right)\cdot \text{PAP}\left(f\right)+Y_{2U}\left(f\right)\cdot H_{3}\left(f\right)\cdot \text{PAP}\left(f\right)+\;Y_{2U}\left(f\right)\cdot {\text{LAP}}_{0}\left(f\right)+Y_{2U}\left(f\right)\cdot N_{\mathrm{LAP}}\left(f\right)+N_{\mathrm{PAF}1}\left(f\right)$$

Thus,

(A14) $$\begin{array}{cc} \text{PAF}\left(f\right)\cdot \text{PAP}{\left(f\right)}^{}* & =Y_{2X}\left(f\right)\cdot \text{PAP}\left(f\right)\cdot \text{PAP}{\left(f\right)}^{}*\\ & +Y_{2U}\left(f\right)\cdot H_{3}\left(f\right)\cdot \text{PAP}\left(f\right)\cdot \text{PAP}{\left(f\right)}^{}*\\ & +\;Y_{2U}\left(f\right)\cdot \text{PAP}{\left(f\right)}^{}*\cdot {\text{LAP}}_{0}\left(f\right)\\ & +Y_{2U}\left(f\right)\cdot \text{PAP}{\left(f\right)}^{}*\cdot N_{\mathrm{LAP}}\left(f\right)\\ & +\text{PAP}{\left(f\right)}^{}*\cdot N_{\mathrm{PAF}1}\left(f\right), \end{array}$$

where PAP(*f*)* is a conjugate complex of PAP(*f*). With the I1O1 analysis, PA admittance [*Y*_*1*_(*f*)] is calculated via ensemble average as the following equation.

(A15) $$Y_{1}\left(f\right)=\frac{E\left[\text{PAF}\left(f\right)\cdot \text{PAP}{\left(f\right)}^{}*\right]}{E\left[\text{PAP}\left(f\right)\cdot \text{PAP}{\left(f\right)}^{}*\right]}$$

Equation A14 gives

(A16) $$Y_{1}\left(f\right)=Y_{2X}\left(f\right)+Y_{2U}\left(f\right)\cdot H_{3}\left(f\right)$$

Hence, PA admittance by the I1O1 analysis, corresponding to a reciprocal of PAZ, can be affected partly by the transfer function from PA to LA pressure.
